# Supplementary material for: First detection of Leptospira santarosai in the reproductive track of a boar: A potential threat to swine production and public health
Source: PLoS One. 2022 Sep 21;17(9):e0274362. doi: 10.1371/journal.pone.0274362 (PMC9491572; doi:10.1371/journal.pone.0274362)
Supplement: S1 Table — (DOCX) [file pone.0274362.s001.docx]

**S1 Table. Microscopic Agglutination Test of infected boar.**

|  |  | **Serovar** | | | | | |
| --- | --- | --- | --- | --- | --- | --- | --- |
| **Date** | **Sample** | **Icterohaemorrhagiae** | **Pomona** | **Canicola** | **Hardjo** | **Grippotyphosa** | **Wolffi** |
| 31/07/2019 | S1 | Negative | Negative | 1:100 | Negative | Negative | Negative |
| 19/08/2019 | S2 | Negative | 1:100 | Negative | 1:100 | Negative | Negative |
