# Supplementary material for: First detection of Leptospira santarosai in the reproductive track of a boar: A potential threat to swine production and public health
Source: PLoS One. 2022 Sep 21;17(9):e0274362. doi: 10.1371/journal.pone.0274362 (PMC9491572; doi:10.1371/journal.pone.0274362)
Supplement: S1 File — (DOCX) [file pone.0274362.s002.docx]

**S1 File. Concatenated consensus sequences of icdA, lipL41, secY, and 16S rDNA gene fragments.**

>Concatenated

CAAGTCAAGCGGAGTAGCAATACTCAGCGGCGAACGGGTGAGTAACACGTGGGTAATCTTCCTTCGAGTCTGGGATAACTTTCCGAAAGGGnAGCTAATACTGGATAGTCCCGAnAGATCATAaGATnTTTCGGGTAAAGATTCATTGCTCGGAGATGAGCCCGCGTCCGATTAGCTAGTTGGTGAGGTAAAGGCTCACCAAGGCGACGATCGGTAGCCGGCCTGAGAGGGTGTTCGGCCACAATGGAACTGAGACACGGTCCATACTCCTACGGGAGGCAGCAGTTAAGAATCTTGCTCAATnnGGGGAACCCTGAAGCAGCGACGCCGCGTGAACGATGAAGGTCTTCGGATTGTAAAGTTCAATAAGCAGGGAAAAATAAGCAGCnATGTGATGATGGTACCTGCCTAAAGCACCGGCTAACTACGTGCCAGCAGCCGCGGTAATACGTATGGTGCAAGCGTTGTTCGGAATCATTGGGCGTAAAGGGTGCGTAGGCGGACATGTAAGTCAGGTGTGAAAACTGCnGGCnCAAGTCCCTTAATTTTAGACTTCTTCATGAAGCCTTCGTAGTTTCTCATCAAAAGTTGAGACTCGATTTGTTTCAAAGTCTCCAGCGCAACCCCTACCATAATxxxxAGxGACGTTCCACCAAACGTATAAACCAAAGATCCACCTCCxGAGTTGGAGCTCAAATCTAAGAATTTGATGATGATGTAAGGAGCCAxxGCCAATCCxGCGAGGAACATCGCGCCGGGAAGTGTGATTCTGTTTAACACxCTTTCGATGTATTCTTTTGTGTGAGAACCxGGACGAATxCCxGGAATGAACCCGCCGTATTTCTTCAGGTTTTCGGACAACxCCGCAGGGTTGAACTGAATCGCAGTxTAAAAGTAxGCxAAGAAAATAATCAAAGAAGTATAGATCACAAAGTAGAACAATGCGTGATACCAGATCTGCGAGAACGGATTxGAAAAGTCCATAATGAxxGCCCAxCCCGCCCACTGTTxxCxGCTGxAAGAxxxxCxCTGAATxATCxTCxGxxGxATAGGAAATTGCGCAGCTACAGTCGATGTAGAATATCCGGTATTCCxGAAAGATAAAGAAGGCCGTGCACTTCAGAAATTCCTCGGAACAATTCGTAACGTxGGTTTGGCGGTCGAACCTCCGAAGAAAAGTCTTTGGGAAGCAATxTTCxGAGAAGGTTCCAG*TTTATCGATCAAATGCCTTCTAAAGTTTTCGAAGCATTCGATAAGGAATCxTATTACAAACTTACCGACCTTAGCAAACGCGCCGA**TATTCAAxGAAGCGACTCTTTCCCTTACGGGAATCACTAAGTCCAGAGCGAAGATCGGAAACCTGATCGGAGCGGAAGCxATTCT*TACATCGGATATCAAAAACC*TATACCGAGTGCAGTACTGAAAACAAAATCGACGCGGTTGCTGCTGGTATCAAAGTAGCGGGTTTTGTGGCTTCTGCGGCGACCGGTAAAGACGTAAACACAGGAAACGATCCTGTTTCAAAACCGACCxGAGTTCGTATGATGTTAATTCCTCTCGATTGAGATCCGCAGCTTTxGxxTTTGCAAGTTCGTCAAAAATCGCGCGGAATCTGGCGTGATATTTTTTGGAAATGGTATxCTTTGTCGCGAACCAAAGATCGATCxTTTCGGAAATCGCGTAATTGAAACAGGCCTGAGCAAAACTCAAAATGGATTTATCCAAGTTATGCTGACCCATGATCACACCTGCTCCGTCGAAGTCGTGGATCAAAGCTCTTTGTTTTTCxTTTCCGTCxTTTCCGGTATAAACGAGTTCCACTTTTCCGGCTTCCGGAATGTAAATTTCGGTGTCCTTATAAAGATCCCCGTAAGCGTGACGTCCGACCGTAATCGCTTTTTTCCAAGAACGAACCGCAGG*GGGATAxTTTTTACTATGATCGGTTTGCGxAAAACGGTTCCGTCCAAAATCGAACGAATCGTTCCGTTCGGAGATTTCCATTCTTGTTTGAGATTGTATTCCTTCACTCTATCTTGGTTCGGAGTGATGGTCGCACATTTTACGCCCACTCCGTATTTTTGAATCGCGTGTGCGGAATCTACGGTAACCTTATCGTCGGTTTTATCCCTATATTCCACGCCTAAATCATAATAGTCCAGTTCGATATCGAGATAAGGATGAACGAACCTATCCTTGATCTCCTTCCAGATGATCCTGGTCATCTCGT
